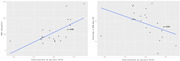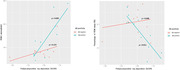# Association Between in vivo Tau Deposition in the Pedunculopontine Nucleus and REM Sleep Alterations in Non‐demented Older Adults

**DOI:** 10.1002/alz.088948

**Published:** 2025-01-09

**Authors:** So Yeon Jeon, Dahyun Yi, Min Soo Byun, Nayeong Kong, Joon Hyung Jung, Yoon Young Chang, Musung Keum, Hyejin Ahn, Jun‐Young Lee, Yun‐Sang Lee, Yu Kyeong Kim, Koung Mi Kang, Chul‐Ho Sohn, Yu Jin Lee, Dong Young Lee, KBASE Research Group

**Affiliations:** ^1^ Chungnam National University Hospital, Daejeon, Daejeon Korea, Republic of (South); ^2^ School of Medicine, Chungnam National University, Daejeon Korea, Republic of (South); ^3^ Institute of Human Behavioral Medicine, Medical Research Center, Seoul National University, Seoul Korea, Republic of (South); ^4^ Seoul National University Hospital, Seoul Korea, Republic of (South); ^5^ Department of Neuropsychiatry, Seoul National University Hospital, Seoul Korea, Republic of (South); ^6^ Department of Psychiatry, Keimyung University Hospital, Daegu Korea, Republic of (South); ^7^ Chungbuk National University Hospital, Cheongju Korea, Republic of (South); ^8^ Interdisciplinary program of cognitive science, Seoul National University, Seoul Korea, Republic of (South); ^9^ Department of Neuropsychiatry, SMG‐SNU Boramae Medical Center, Seoul Korea, Republic of (South); ^10^ Department of Nuclear Medicine, Seoul National University College of Medicine, Seoul Korea, Republic of (South); ^11^ Seoul National University College of Medicine, Seoul Korea, Republic of (South); ^12^ SMG‐SNU Boramae Medical Center, Seoul Korea, Republic of (South); ^13^ Department of Radiology, Seoul National University Hospital, Seoul Korea, Republic of (South); ^14^ Department of Psychiatry, Seoul National University College of Medicine, Seoul Korea, Republic of (South)

## Abstract

**Background:**

Rapid eye movement(REM) sleep alterations are increasingly recognized as an early manifestation of Alzheimer's disease as well as Lewy body disease. The pedunculopontine nucleus(PPN), the main brain source of cholinergic innervation, is known to play an important role in the regulation of REM sleep and has been reported to be one of the early brainstem regions where hyperphosphorylated tau accumulates. Nevertheless, little information is available about the relationship between tau pathology in the PPN(PPN‐tau) and REM sleep disturbance. Therefore, we aimed to investigate the relationship between Tau‐PPN and REM sleep characteristics, alongside the modulating role of amyloid pathology.

**Method:**

A total of 22 non‐demented older adults from the Korean Brain Aging Study for Early Diagnosis and Prediction of Alzheimer’s Disease were included. All participants underwent [^11^C]‐PiB PET and [^18^F]‐AV1451‐PET. Sleep variables were measured through nocturnal polysomnography(PSG) over two consecutive nights at a center. Tau deposition in the PPN was measured in the ROI masks for each participant, prepared in native space using the Harvard arousal network atlas for the PPN. Linear regression analyses were performed with sleep variables as dependent variables and tau deposition levels as independent variables after controlling for age, sex, education, and apolipoprotein‐E ε4 positivity. In an exploratory analysis, sleep variables demonstrating significance at a threshold of p < 0.1 were further subjected to subgroup analysis based on amyloid positivity.

**Result:**

PPN‐tau was significantly correlated with increased REM latency (p<0.001; Figure 1) and showed a trend‐level association with REM sleep percentage(REM %) (p=0.079). Even after controlling for global tau deposition and amyloid positivity, the significance did not change (p = 0.014). In addition, subgroup analyses according to Aβ positivity revealed that higher PPN‐tau was significantly related to increased REM latency (p = 0.001) and decreased REM % (p=0.011) in Aβ‐positive group but not in Aβ‐negative group (p=0.123 for REM latency and p=0.690 for REM %) (Figure 2).

**Conclusion:**

Our findings support the possibility that tau pathology in the PPN underlies REM sleep alteration, more specifically, increased REM latency and decreased REM sleep in non‐demented older adults. Additionally, this relationship appears to be more prominent in individuals with brain Aβ pathology.